# Sex differences in associations among metabolic syndrome, obesity, related biomarkers, and colorectal adenomatous polyp risk in a Japanese population

**DOI:** 10.3164/jcbn.18-11

**Published:** 2018-04-03

**Authors:** Keisuke Nakai, Jiro Watari, Katsuyuki Tozawa, Akio Tamura, Ken Hara, Takahisa Yamasaki, Takashi Kondo, Tomoaki Kono, Toshihiko Tomita, Yoshio Ohda, Tadayuki Oshima, Hirokazu Fukui, Jun Sakurai, Yongmin Kim, Yuji Hayakawa, Takashi Fujisawa, Takeshi Morimoto, Hiroto Miwa

**Affiliations:** 1 Division of Gastroenterology, Department of Internal Medicine, Hyogo College of Medicine, 1-1 Mukogawa-cho, Nishinomiya, Hyogo 663-8501, Japan; 2 Department of Gastroenterology, Meiwa Hospital, 3-39 Kaminaruo-cho, Nishinomiya, Hyogo 663-8186, Japan; 3 Department of Gastroenterology, Steel Memorial Hirohata Hospital, 3-1 Yumesaki-cho, Hirohata-ku, Himeji, Hyogo 671-1122, Japan; 4 Department of Clinical Epidemiology, Hyogo College of Medicine, 1-1 Mukogawa-cho, Nishinomiya, Hyogo 663-8501, Japan

**Keywords:** sex difference, metabolic syndrome, visceral obesity, colorectal adenoma, adipokine

## Abstract

To investigate sex differences in the associations among metabolic syndrome, obesity, adipose tissue-related biomarkers, and colorectal adenomatous polyps, a cross-sectional, multicenter study was conducted on 489 consecutive individuals who underwent their first colonoscopy at 3 hospitals. Plasma concentrations of adiponectin and leptin, as well as homeostatic model assessment of insulin resistance were also evaluated. The presence and number of adenomatous polyps, including advanced adenoma, were higher in men than in women. Metabolic syndrome was a risk factor for adenomatous polyps in both sexes. Large waist circumference was an independent risk factor for adenomatous polyps in men, and high BMI and large waist circumference were risk factors for adenomatous polyps in women. Interestingly, low BMI was associated with large adenomatous polyps (≥10 mm) and advanced adenoma, and waist-hip ratio was involved in proximal adenomatous polyp development only in women. In contrast, the highest quartile of leptin concentration had a 3.67-fold increased adenomatous polyp risk compared with the lowest quartile only in men. These results indicate that regarding colorectal pathogenesis, sex differences were identified in obesity but not in metabolic syndrome. Visceral obesity and a high serum leptin level may be risk factors for colorectal adenomatous polyp development in Japanese men.

## Introduction

The incidence and prevalence of metabolic syndrome (MetS) is rising in worldwide.^(1)^ MetS, which is characterized by central obesity, high blood pressure, hyperglycemia, and dyslipidemia,^(2)^ have been reported to increase the risk not only for cardiovascular disease,^(3,4)^ but also for cancer.^(5–7)^ The prevalence of obesity is certainly increasing in Asian countries because of the adoption of Western dietary habits and lifestyle.^(8,9)^

The mortality rate of colorectal cancer (CRC) has been increasing, and CRC was recently found to be the third-most-common cancer among men and the most common cancer among women in Japan.^(10)^ Therefore, early detection and removal of colorectal adenomatous polyps (APs) are essential for reducing the mortality rate of CRC.^(11,12)^ To date, many studies on the associations of MetS and obesity with colorectal neoplasms, including APs and CRCs, have been reported in Asian populations.^(13–22)^ According to a meta-analysis, an increase of 5 kg/m^2^ in body mass index (BMI) in men confers a relative risk of 1.24 for CRC.^(23)^ However, in women, the association between BMI and the risk of CRC is complicated by their difference in fat distribution compared with men. Indeed, a pooled analysis showed sex-associated differences in the risk of CRC by waist circumference (WC) and waist-hip ratio (WHR).^(24)^

Visceral adipose tissue is more metabolically active than subcutaneous fat, and produces adipokines, such as adiponectin and leptin, that can lead to proinflammatory, procoagulant, and insulin-resistant states, which may promote tumorigenesis in the local environment.^(25)^ The importance of adipose tissue location in terms of the risk of metabolic dysfunction is evident because central obesity is more strongly associated with an increased risk of insulin resistance than BMI alone.^(26,27)^

To date, although several studies on the associations of obesity, adipokines, leptin, and insulin resistance with colorectal adenomatous polyps (APs) in a Japanese population^(14,28–31)^ have been reported, there are no prospective studies on the sex differences in Japanese subjects who have never undergone colonoscopy. The current multicenter, hospital-based, cross-sectional study assessed the associations among MetS, obesity, adipose tissue-associated biomarkers, and colorectal APs as a precancerous condition.

## Materials and Methods

### Patients

Between February 2014 and December 2016, 489 consecutive individuals who underwent a first colonoscopy at the Gastroenterology Division of Hyogo College of Medicine Hospital (*n* = 427), Meiwa Hospital (*n* = 12), and Steel Memorial Hirohata Hospital (*n* = 50) were enrolled in this study. Most patients were outpatients; those who had previously undergone colorectal surgery or colonoscopy were excluded. At the participating facilities, colonoscopy was performed for various reasons, such as a positive fecal occult blood test (*n* = 192, 39.3%), detailed examination of abdominal symptoms (*n* = 162, 33.1%), medical check-up (*n* = 70, 14.3%), and other reasons. A standardized questionnaire was used to obtain data from each subject regarding family history of CRC and smoking and alcohol habits prior to endoscopy. Whether subjects were regularly taking non-steroidal anti-inflammatory drugs (NSAIDs) or low-dose aspirin (LDA) for more than one year was determined by self-reporting and by a review of the medical prescriptions in the database. The Ethics Committee of Hyogo College of Medicine and the participating hospitals approved this study (No. 1654). Written, informed consent was obtained from all individuals prior to this study. This trial was registered with the UMIN Clinical Trials Registry (number UMIN000018044). The study was performed in accordance with the Declaration of Helsinki.

### Definition of the metabolic syndrome

According to proposal of the Japanese Committee for the Diagnostic Criteria of Metabolic Syndrome,^(32)^ MetS was defined as a combination of abdominal obesity as indicated by a large waist circumference (WC) (men, ≥85 cm; women, ≥90 cm) with any two of the following three conditions: 1) elevated triglycerides (≥150 mg/dl) and/or lowered high-density lipoprotein-cholesterol (HDL-Cho) (<40 mg/dl); 2) elevated blood pressure (BP) (systolic BP ≥130 mmHg and/or diastolic BP ≥85 mmHg); and 3) high serum fasting blood sugar (FBS) (≥110 mg/dl). Medication for hypertension and treatment for diabetes mellitus (DM) were taken as evidence of raised BP and FBS, respectively.

### Anthropometric measurements

Prior to endoscopy, the following body measurements were performed. BMI was calculated as weight divided by the square of height (kg/m^2^). Obesity was defined as a BMI ≥25.0 kg/m^2^, based on the criteria of the Japanese Society of Obesity.^(33)^ A large WC based on the Japanese criteria (≥85 cm for men, ≥90 cm for women) was defined as an abnormal WC.^(32)^ The high-risk category for WHR was defined as ≥0.9 for men and ≥0.85 for women, in accordance with the definition of Ilanne-Parikka *et al.*^(34)^

### Endoscopy protocol and histology

All individuals underwent narrow-band imaging (NBI) or blue-laser imaging (BLI) endoscopy (endoscope: CF-H260AZI, -HQ290I, -HQ290L, -Q260AI, PCF-Q260AZI, -H290I, and -PQ260L; EC-L590WM and -L590ZP) with an electronic endoscopic system (Elite CV-290, Olympus Medical Systems, Corp., Tokyo, Japan; LASEREO, FUJIFILM Holdings Corp, Tokyo, Japan). The lesion size was judged by comparison with biopsy forceps (the closed forceps were 2.2 mm in diameter, and the open forceps were about 8 mm in diameter; Radial Jaw™ 4: Boston Scientific Corporation, Marlborough, MA). Histological diagnoses of the lesions were made based on the revised Vienna classification and the World Health Organization classification.^(35,36)^ Advanced neoplasms were defined as lesions larger than 10 mm in diameter, lesions with a villous component, and/or with high-grade dysplasia. Lesions histologically diagnosed as categories 3 and 4 were analyzed in this study. CRC in this study was defined as a lesion with submucosal invasion by carcinoma (category 5) and a pathological depth of pT2 or greater (muscularis propria invasion or deeper). In cases that were not diagnosed histologically, lesions that were diagnosed as “type 2” based on the NBI International Colorectal Endoscopic (NICE) classification^(37)^ using NBI or BLI without magnification were judged as adenomas. Lesions in the cecum, ascending colon, and transverse colon were classified as proximal, and those in the descending colon, sigmoid, and rectum were classified as distal.

### Laboratory tests

Serum levels of high-molecular-weight (HMW) adiponectin and insulin were quantified using the CLEIA kit (Fujirebio, Inc. Tokyo, Japan) on a fully-automated system (Lumipulse G1200 or Presto II; Fujirebio, Inc.). Serum leptin levels were measured using a human leptin radioimmunoassay kit (Merck Millipore Co., Darmstadt, Germany). All analyses were assayed at SRL Inc. (Hachioji, Tokyo, Japan). Samples from matched sets were assayed together. All laboratory personnel were blinded with respect to case or control status. The inter-assay coefficients of variation from the quality control samples were 3.3–7.3% for HMW-adiponectin, 7.3–10.8% for leptin, and 2.5–3.9% for insulin. The homeostatic model assessment of insulin resistance (HOMA-IR) was calculated using the following equation: fasting serum glucose (mg/dl) X insulin (mU/L)/405.

### Statistical analysis

Continuous and categorical data are reported as mean ± SD and frequencies with proportions, respectively. The data were compared between the two groups by the Mann-Whitney *U* test for patients’ age and adiposity measures, and the chi-squared test or Fisher exact test for the other variables. Correlations among variables were estimated by Pearson correlation coefficients. Predictive factors with a *p* value of less than 0.1 on univariate analysis were included in the multiple logistic regression model and analyzed using the backward approach. Odds ratios (ORs) and 95% confidence intervals (CIs) were calculated for the risk factors. The 95% CI of the OR was used to assess the statistical significance at the conventional level of 0.05. A *p* value of less than 0.05 was considered to indicate a statistically significant difference between two groups. Statistical analyses were performed with SPSS 22.0 software (SPSS Inc., Chicago, IL).

## Results

### Patient characteristics

Patients’ baseline characteristics are shown in Table 1. Out of 489 individuals who underwent a first colonoscopy, 22 cases (4.5%) were diagnosed as CRC. In the present study, as the numbers of CRCs, sessile serrated adenomas (SSAs), and traditional serrated adenomas (TSAs) were small, patients with those lesions were excluded from this study. Finally, a total of 460 individuals (246 men and 214 women) were enrolled in this study. There was no significant difference in the mean age between the sexes. The incidences of smoking and alcohol drinking were significantly higher in men than in women (*p*<0.0001, each), while that of patients who were taking NSAIDs or LDA or had a family history of CRC was not different between the sexes. APs were more frequently identified in men (55.0%) than in women (38.3%) (*p* = 0.0002), and advanced adenomas were also found significantly more frequently in men (20.2%) than in women (12.8%) (*p* = 0.03). The prevalence of MetS was 21.7% in enrolled patients, and MetS was significantly more frequently observed in men (31.3%) than in women (10.6%) (*p*<0.0001). Among the components of MetS, the frequencies of all components, i.e., large WC, hypertension, dyslipidemia, and DM, were significantly higher in men than in women (*p*<0.0001, *p* = 0.0006, *p* = 0.002, and *p* = 0.03, respectively). Serum levels of TG, HDL-Cho, and FBS were also significantly higher in men than in women (*p*<0.0001, each).

Although the plasma adiponectin concentrations were significantly lower in men than in women (*p*<0.0001), the leptin levels were conversely higher in men than in women (*p*<0.0001). HOMA-IR levels were not significantly different between the sexes.

### Factors predicting development of APs in men and women

#### 1) Predictive factors in men

The mean age was significantly higher in individuals with APs than in those without APs (*p*<0.0001). The frequency of alcohol drinking tended to be higher in subjects with APs than in those without, but the differences did not reach significance (*p* = 0.07). MetS was more frequently identified in individuals with APs than in those without (*p* = 0.001) (Table 2).

Among the components of MetS, large WC, hypertension, and dyslipidemia were associated with APs (*p* = 0.002, *p* = 0.06 and *p* = 0.007, respectively) (Table 3a). However, after adjusting for age (≥65 years) and alcohol drinking on multivariate logistic regression analysis, only large WC was a risk factor for AP (adjusted OR = 2.17, 95% CI = 1.21–3.89, *p* = 0.009). In addition, the adjusted OR for AP increased as the number of components of MetS increased (*p* trend = 0.0002).

#### 2) Predictive factors in women

The mean age was significantly higher in individuals with APs than in those without APs (*p*<0.0001) as in men (Table 2). Although all components of MetS were significantly involved in APs, TG, HDL-Cho, and FBS levels were not different between individuals with and without APs. On multivariate logistic regression analysis, only WC in MetS was significantly associated with age (≥65 years)-adjusted OR for AP (adjusted OR = 3.74, 95% CI = 1.69–8.31, *p* = 0.001), and was an independent risk factor for AP (Table 3b). In addition, the adjusted OR for AP increased as the number of components of MetS increased if at least one of the MetS criteria was met (*p* trend<0.0001).

### Association among MetS, obesity and the clinicopathological features of Aps

In men, the number of APs and advanced adenomas tended to be higher in subjects with MetS than in those without MetS after adjusting for age and alcohol drinking (*p* trend = 0.05 and *p* = 0.07, respectively), whereas there were no significant differences. The size and location of APs were not significantly associated with MetS. In women, all clinicopathological features of APs were not involved in MetS as in men (Table 4).

As only large WC in MetS was significantly associated with a risk of AP in both sexes from the above results (Table 3), the association between obesity and AP was additionally evaluated. In men, large WC and WHR were significantly associated with APs (*p* = 0.002 and *p* = 0.02, respectively), but BMI was not. Multivariate logistic regression analysis demonstrated that a large WC was significantly associated with age (≥65 years) and alcohol intake-adjusted OR for AP (adjusted OR = 2.55, 95% CI = 1.32–4.93, *p* = 0.005), and was an independent risk factor for AP (Table 5a). In women, all obesity measurements were significantly higher in individuals with APs than in those without APs. On multivariate logistic regression analysis that adjusted for age (≥65 years), a high BMI (adjusted OR = 2.74, 95% CI = 1.05–7.13, *p* = 0.04) and large WC (adjusted OR = 3.21, 95% CI = 1.23–8.40, *p* = 0.02) were risk factors for APs (Table 5b).

The number of APs and histological progression from low-grade adenoma to advanced adenoma were not significantly associated with obesity measures in both sexes (Tables 6, 7, and 8). However, AP size of greater than 10 mm and advanced adenoma was associated with a low BMI (*p* = 0.02 and *p* = 0.05, respectively) in women (Table 6). In addition, AP was more frequently identified in the proximal colon than in the distal colon in women with high WHR (*p* = 0.04) (Table 8).

### Associations among obesity, adipose tissue-associated biomarkers, and Aps

Leptin concentration and HOMA-IR level were positively correlated with all anthropometric measurements, such as BMI, WC, and WHR, whereas plasma HMW adiponectin showed a negative correlation with these measurements (Supplemental Table 1*). Individuals in the highest quartile of leptin concentrations had increased adenoma risk compared with those in the lowest quartile in men (3.67-fold, 95% CI = 1.30–10.3, *p* = 0.01), but not in women (Table 9). However, no significant associations between plasma HMW adiponectin or HOMA-IR level and AP were seen in both sexes. The incidence of advanced adenoma was significantly higher in individuals in the highest quartile of HMW adiponectin levels compared with those in the lowest quartile in women (*p* = 0.02) (Supplemental Table 2*). In other cases, the concentrations of adipokines and the HOMA-IR index were not associated with polyp size, polyp number, location, and histology (Supplemental Table 2 a, b, and c*).

## Discussion

To date, there are many reports from Asia regarding the association between MetS or obesity and AP,^(13–22)^ but studies on sex differences in the association are limited.^(13,15,20)^ The present study clearly demonstrated that individuals with MetS had increased adenoma risk compared with those without MetS in both sexes (2.03-fold in men and 8.40-fold in women). Additionally, among the components of MetS, only large WC was an independent risk factor for AP in both sexes.

Our results were in agreement with several previous studies in Asia;^(13,20)^ however, Liu *et al.*^(18)^ reported finding the association between MetS and AP more strongly in men than in women. There are some possible explanations for the discrepancy; differences of sample size analyzed, the definition of MetS, and ethnic difference may have affected the results. Although we used the Japanese criteria^(32)^ as the definition of MetS in this study, other studies adopted the definitions of the modified National Cholesterol Education Program Adult Treatment Panel III (ATP III) for Asians,^(38)^ the America Heart Association and National Heart Lung Blood Institute (AHA/NHLBI),^(39)^ or the International Diabetes Federation (IDF).^(40)^ Therefore, it may be impossible to make direct comparisons between data from studies when different definitions have been used to identify subjects with the syndrome.^(41)^ Even in Asian countries, the cut-points for WC as central obesity are different.^(41)^ Importantly, the AHA/NHLBI scientific statement emphasized that large WC is an important risk in MetS in Asian populations.^(39)^ In addition, the new IDF definition differs from the ATP III definition in that it requires evidence of central obesity for the diagnosis of MetS.^(41)^ The rationale for this requirement is that central obesity is the parameter showing the strongest correlation than other MetS features.^(42,43)^ Therefore, our result was consistent with these directives.

Next, we investigated the association between obesity and AP because only WC in MetS was a risk factor of AP. All body measurements were significantly higher in men than in women. Until now, there have been several reports from Japan regarding the association.^(14,28–31,44,45)^ However, the association between obesity and AP still remains controversial. Intriguingly, recent Mendelian randomization analyses showed that BMI was significantly associated with CRC in women, but the association of WHR with CRC was only significant for men;^(46,47)^ these findings are in agreement with the present results if AP is considered to be a precancerous lesion of CRC.

To date, reports from Asia, including Japan, have demonstrated that MetS and obesity were associated with right-colon AP, advanced adenomas and multiple APs.^(14–16,18,19,28)^ In our study, the number of APs and advanced adenomas tended to be higher only in men with MetS but not in women with MetS. Interestingly, APs were observed at a significantly higher rate in the proximal colon than in the distal colon in women with visceral obesity, as indicated by a high WHR, thus supporting the previous studies that obesity was strongly associated with proximal AP.^(14,15)^ However, it remains unclear as to why visceral obesity was involved in proximal AP development and a low BMI was associated with large APs and advanced adenoma only in women. APs ≥10 mm and advanced adenomas were significantly associated with low BMI in women, which was different from previous studies.^(14,15,28)^ It might be difficult to explain the discrepancy and mechanisms. Nam *et al.*^(48)^ reported that sex differences in the association of MetS and its individual components with adenomas may be explained by menstrual status in addition to differential patterns of visceral fat deposition. Thus, some factors other than MetS and obesity may be involved in the growth but not emergence of APs. Further investigations will be required on a larger sample size to more clearly confirm the relationship between these associations.

In the present study, adipose tissue-associated biomarkers were significantly correlated with all anthropometric measurements. Visceral adipose tissue secretes a number of adipokines and cytokines, such as leptin, tumor necrosis factor-α, and IL-6, leading to a proinflammatory and insulin-resistant state.^(49)^ This phenomenon causes the decreased release of anti-inflammatory adipokines, including adiponectin.^(50)^ An association between colorectal neoplasms and these inflammatory cytokines has been postulated, in which the increased production of proinflammatory cytokines and decreased production of anti-inflammatory adiponectin in adipocytes may be associated with the risk of AP and CRC.^(31,51–53)^ It has also been reported that plasma adiponectin levels, particularly HMW adiponectin analyzed in this study, is a convenient and sensitive biomarker for the prediction of insulin resistance (HOMA-IR).^(54)^ However, the role of these biomarkers, including insulin resistance in the development of APs remains unclear, as previously reported.^(28,31,55–60)^ In the current study, only the serum leptin level, not HMW adiponectin and HOMA-IR, was associated with the presence of AP in men, but not in women. Although there are several studies including a meta-analysis that reported the association between serum leptin concentration and AP,^(31,44,56–61)^ the results were inconsistent. The results of two studies that analyzed the sex differences in the associations between the conditions were similar to our results.^(44,59)^ In addition, when analyzing the associations between adipose tissue-related biomarkers and the number, size, and histology of APs, there were no significant differences in men. However, a polyp size ≥10 mm was associated with high levels of HMW adiponectin in women. This result was different from the previous studies, in which decreased serum adiponectin is significantly involved in the development of colorectal adenoma.^(28,30,31,55)^ On the other hand, some studies have shown that no association between adiponectin levels and colorectal adenoma were found.^(57,58)^ Taking the findings of these reports and the present findings into consideration, data regarding the effect of the biomarkers on AP development may be contradictory and difficult to interpret.^(25,57)^

In the present study, there was a methodological advantage, as we analyzed data only from individuals who had undergone their first colonoscopy. Beyond this advantage, our study has some limitations. One possible weakness is that the sample size, particularly the number of asymptomatic individuals, was relatively small. However, most subjects who showed a positive fecal occult blood test were asymptomatic, and the symptoms in most subjects who underwent a detailed examination of abdominal symptoms were transient. Second, some APs found during colonoscopy could not be diagnosed histologically. However, the NICE classification used in this study holds promise for meeting the standard minimum performance benchmarks for real-time endoscopic assessment of histology even in diminutive colorectal polyps because of its high accuracy, sensitivity, specificity, and positive and negative predictive values for the diagnosis of adenomatous lesions.^(37)^ Third, laboratory tests could not be obtained from all individuals enrolled because they were non-fasting at the time of blood collection. However, the number of samples in our study was considered to be sufficient to detect significant differences in each biomarker with respect to colorectal APs. Fourth, we excluded patients with CRC, SSA and TSA due to the limited number of such cases; also, the catabolic effect of CRC would affect the components of MetS and amounts of adipose tissue.^(62)^ Therefore, only the association among APs as precancerous lesions, MetS and obesity was investigated.

In conclusion, the AP risk increased with the number of MetS components in both sexes. In contrast, the risk factors for AP were different between men and women from the viewpoint of obesity and the adipose tissue-related biomarker, leptin. Recently, a new anthropometric measure, a body shape index (ABSI) has been suggested to be superior to BMI and WC as a measure of disease risk, in particular for premature mortality.^(63,64)^ Therefore, further large studies in populations with larger body sizes on how these associations vary by sex and obesity including ABSI in Asia are required.

## Author Contributions

Watari J, Morimoto T, and Miwa H designed and supervised the study; Nakai K, Watari J, and Morimoto T contributed to the statistical analysis; Watari J and Nakai K contributed to the writing of the draft manuscript; Nakai K, Watari J, Tozawa K, Tamura A, Hara K, Yamasaki T, Kondo T, Kono T, Tomita T, Ohda Y, Ohshima T, Fukui H, Sakurai J, Kim Y, Hayakawa Y, Fujisawa T, and Miwa H collected the data; Morimoto T and Miwa H made the critical revision of the manuscript. All authors discussed the results and commented on the manuscript.

## Figures and Tables

**Table 1:** Patient characteristics

Categorical variables	Total (*n* = 489)	Men (*n* = 262)	Women (*n* = 227)	*p* (men vs women)
Mean ± SD age (years)	59.0 ± 15.7	60.1 ± 15.7	57.8 ± 15.6	0.1
Current smoking	80 (16.4)	61 (23.3)	19 (8.4)	<0.0001
Alcohol drinking	125 (25.6)	106 (40.5)	19 (8.4)	<0.0001
NASIDs/LDA use	70 (14.3)	33 (12.6)	37 (16.3)	0.24
Family history of CRC	62 (12.7)	27 (10.3)	35 (15.4)	0.09
Prevalence				
AP	231 (47.2)	144 (55.0)	87 (38.3)	0.0002
(Advanced Adenoma)	82 (16.8)	53 (20.2)	29 (12.8)	0.03
SSA and TSA	7 (1.4)	3 (1.1)	4 (1.8)	0.57
CRC	22 (4.5)	13 (5.0)	9 (4.0)	0.67
MetS	106 (21.7)	82 (31.3)	24 (10.6)	<0.0001
WC (cm)	84.1 ± 11.1	87.4 ± 9.6	80.3 ± 11.6	<0.0001
Hypertension	177 (23.9)	113 (43.1)	64 (28.2)	0.0006
Dyslipidemia	216 (44.1)	133 (50.8)	83 (36.6)	0.002
Diabetes mellitus	72 (14.7)	47 (17.9)	25 (11.0)	0.03
TG (mg/dl)	126.9 ± 90.8	140.2 ± 97.3	111.5 ± 80.2	<0.0001
HDL-Cho (mg/dl)	59.0 ± 20.5	53.7 ± 20.2	65.0 ± 19.2	<0.0001
FBS (mg/dl)	108.5 ± 34.7	112.7 ± 41.3	103.6 ± 24.3	<0.0001
Adiponectin (µg/ml)	5.1 ± 3.6	4.0 ± 2.9	6.3 ± 3.9	<0.0001
Leptin (ng/ml)	11.5 ± 12.1	9.0 ± 12.3	14.1 ± 11.3	<0.0001
HOMA-IR	2.37 ± 3.67	2.63 ± 4.27	2.10 ± 2.92	0.26

NSAID, non-steroidal anti-inflammatory drugs; LDA, low-dose aspirin; AP, adenomatous polyp; SSA, sessile serrated adenoma; TSA, traditional serrated adenoma; CRC, colorectal cancer; WC, waist circumference; MetS, metabolic syndrome; TG, triglyceride; HDL-Cho, high-density lipoprotein cholesterol; FBS, fasting blood sugar; HOMA-IR, homeostatic model assessment of insulin resistance.

**Table 2:** Comparison of patient characteristics with and without adenomatous polyps

Categorical variables	Men				Women		
	Individuals with APs (*n* = 144)	Individuals without APs (*n* = 102)	*p*		Individuals with APs (*n* = 87)	Individuals without APs (*n* = 127)	*p*
Mean age ± SD (years)	64.8 ± 12.7	51.6 ± 16.5	<0.0001		63.7 ± 13.0	52.8 ± 15.9	<0.0001
Current smoking	34 (23.6)	24 (23.5)	0.99		5 (5.7)	12 (9.4)	0.33
Alcohol drinking	63 (43.8)	33 (32.4)	0.07		7 (8.0)	10 (7.9)	0.96
Family history of CRC	18 (12.5)	8 (7.8)	0.24		16 (18.4)	15 (11.8)	0.18
NASIDs/LDA use	21 (14.6)	11 (10.8)	0.38		16 (18.4)	18 (14.2)	0.41
MetS	54 (37.5)	19 (18.6)	0.001		19 (21.8)	3 (2.4)	<0.0001
WC (cm)	88.7 ± 8.7	84.9 ± 10.6	0.0003		84.6 ± 14.2	77.3 ± 8.7	<0.0001
Hypertension	67 (46.5)	35 (34.3)	0.06		37 (38.1)	23 (18.1)	<0.0001
Dyslipidemia	83 (57.6)	41 (40.2)	0.007		42 (48.3)	33 (26.0)	0.0008
Diabetes mellitus	27 (18.8)	12 (17.7)	0.14		16 (18.4)	6 (4.7)	0.002
TG ≥150 (mg/dl)	57 (39.6)	24 (23.5)	0.008		20 (23.0)	22 (17.3)	0.3
HDL-Cho <40 (mg/dl)	33 (22.9)	16 (15.7)	0.16		3 (3.4)	11 (8.7)	0.16
FBS ≥110 (mg/dl)	52 (36.1)	36 (35.3)	0.9		20 (23.0)	22 (17.3)	0.31

CRC, colorectal cancer; NSAID, non-steroidal anti-inflammatory drug; LDA, low-dose aspirin; MetS, metabolic syndrome; WC, waist circumference; TG, triglyceride; HDL-Cho, high-density lipoprotein cholesterol; FBS, fasting blood sugar.

**Table 3:** Predictors of the development of adenomatous polyp in individuals with MetS

a. Men									
Categorical variables	Individuals with APs (*n* = 144)	Individuals without APs (*n* = 102)	Univariate analysis			Multivariate analysis			
			OR (95% CI)	*p*		OR (95% CI)	*p*	OR (95% CI)*	*p*

Age (years)									
<65	63 (43.8)	73 (71.6)	1						
65≤	81 (56.2)	29 (28.4)	3.24 (1.88–5.56)	<0.0001					
MetS									
No	90 (62.5)	83 (81.4)	1					1	
Yes	54 (37.5)	19 (18.6)	2.62 (1.44–4.79)	0.001				2.03 (1.08–3.82)	0.03
WC (cm)									
<85	46 (31.9)	53 (52.0)	1			1		1	
85≤	98 (68.1)	49 (48.0)	2.30 (1.37–3.89)	0.002		1.90 (1.10–3.31)	0.02	2.17 (1.21–3.89)	0.009
Hypertension									
No	77 (53.5)	67 (65.7)	1			1		1	
Yes	67 (46.5)	35 (34.3)	1.67 (0.99–2.81)	0.06		1.36 (0.78–2.35)	0.28	0.84 (0.46–1.55)	0.58
Dyslipidemia									
No	61 (42.4)	61 (59.8)	1			1		1	
Yes	83 (57.6)	41 (40.2)	2.02 (1.21–3.39)	0.007		1.61 (0.93–2.78)	0.09	1.46 (0.82–2.58)	0.2
Diabetes mellitus									
No	117 (81.2)	90 (82.3)	1						
Yes	27 (18.8)	12 (17.7)	1.73 (0.83–3.60)	0.14					
Number of MetS components									
0	19 (13.2)	30 (29.4)	1					1	
1	39 (27.1)	26 (25.5)	2.37 (1.11–5.06)	0.02				1.93 (0.86–4.34)	0.11
2	35 (24.3)	31 (30.4)	1.78 (0.84–3.78)	0.13				1.65 (0.77–3.54)	0.2
3	39 (27.1)	12 (11.8)	5.13 (2.16–12.2)	0.0001				3.81 (1.48–9.82)	0.006
4	12 (8.3)	3 (2.9)	6.32 (1.57–25.4)	0.007				4.83 (1.08–21.6)	0.04
*p* trend				0.0002					


b. Women																		
Categorical variables	Individuals with APs (*n* = 87)	Individuals without APs (*n* = 127)	Univariate analysis			Multivariate analysis			
			OR (95% CI)	*p*		OR (95% CI)	*p*	OR (95% CI)^†^	*p*

Age (years)									
<65	37 (42.5)	91 (71.7)	1						
65≤	50 (57.5)	36 (28.3)	3.42 (1.92–6.07)	<0.0001					
MetS									
No	68 (78.2)	124 (97.6)	1					1	
Yes	19 (21.8)	3 (2.4)	11.6 (3.30–40.5)	<0.0001				8.40 (2.34–30.2)	0.001
WC (cm)									
<90	55 (63.2)	115 (90.6)	1			1		1	
90≤	32 (36.8)	12 (9.4)	5.58 (2.67–11.7)	<0.0001		3.84 (1.74–8.46)	0.001	3.74 (1.69–8.31)	0.001
Hypertension									
No	50 (61.9)	104 (81.9)	1			1		1	
Yes	37 (38.1)	23 (18.1)	3.35 (1.80–6.22)	<0.0001		1.85 (0.91–3.76)	0.09	1.37 (0.64–2.91)	0.42
Dyslipidemia									
No	45 (51.7)	94 (74.0)	1			1		1	
Yes	42 (48.3)	33 (26.0)	2.66 (1.49–4.74)	0.0008		1.82 (0.95–3.51)	0.07	1.76 (0.91–3.42)	0.09
Diabetes mellitus									
No	71 (81.6)	121 (95.3)	1			1		1	
Yes	16 (18.4)	6 (4.7)	4.55 (1.70–12.2)	0.002		2.53 (0.87–7.35)	0.09	2.23 (0.77–6.49)	0.14
Number of MetS components									
0	23 (26.4)	78 (61.4)	1					1	
1	26 (29.9)	27 (21.3)	3.27 (1.60–6.65)	0.0009				2.80 (1.32–5.96)	0.007
2	17 (19.5)	19 (15.0)	3.03 (1.36–6.77)	0.006				2.34 (0.97–5.61)	0.06
3	17 (19.5)	3 (2.3)	19.2 (5.17–71.4)	<0.0001				20.3 (4.91–84.0)	<0.0001
4	4 (4.7)	0 (0.0)	30.1 (1.56–579.4)	0.004				—	1
*p* trend				<0.0001					

*Adjusted for age (≥65 years) and alcohol drinking. ^†^Adjusted for age (≥65 years). MetS, metabolic syndrome; WC, waist circumference.

**Table 4:** MetS and adenomatous polyps

Categorical variables	Men					Women			
	MetS+ (*n* = 54)	MetS– (*n* = 90)	OR (95% CI)	*p*		MetS+ (*n* = 19)	MetS– (*n* = 68)	OR (95% CI)	*p*
Number									
1	13 (24.1)	30 (33.3)	1			11 (58.0)	32 (47.1)	1	
2	9 (16.7)	24 (26.7)	0.86 (0.31–2.35)	0.76*		4 (21.0)	20 (29.4)	0.50 (0.13–1.89)	0.31^†^
3≤	32 (59.2)	36 (40.0)	1.84 (0.81–4.22)	0.15*		4 (21.0)	16 (23.5)	0.65 (0.32–1.30)	0.22^†^
*p* trend				0.05					0.53
Size (mm)									
<5	14 (25.9)	19 (21.1)	1			7 (36.8)	18 (26.5)	1	
5≤ <10	15 (27.8)	44 (48.9)	0.41 (0.16–1.04)	0.06*		8 (42.2)	27 (39.7)	0.69 (0.20–2.35)	0.55^†^
10≤	25 (46.3)	27 (30.0)	0.95 (0.37–2.45)	0.92*		4 (21.0)	23 (33.8)	0.55 (0.26–1.15)	0.11^†^
*p* trend				0.38					0.25
Location									
Proximal colon	29 (53.7)	40 (44.4)	1			10 (52.6)	32 (47.1)	1	
Distal colon	25 (46.3)	50 (55.6)	0.63 (0.31–1.27)	0.19*		9 (47.4)	36 (52.9)	1.09 (0.37–3.22)	0.87^†^
Advanced adenoma									
No	28 (51.8)	63 (70.0)	1			15 (79.0)	43 (63.2)	1	
Yes	26 (48.2)	27 (30.0)	1.95 (0.95–4.02)	0.07*		4 (21.0)	25 (36.8)	0.33 (0.09–1.15)	0.08^†^

MetS, metabolic syndrome. *Adjusted for age (≥65 years) and alcohol drinking. ^†^Adjusted for age (≥65 years).

**Table 5:** Predictors of the development of adenomatous polyp

a. Men									
Categorical variables	Individuals with APs (*n* = 144)	Individuals without APs (*n* = 102)	Univariate analysis			Multivariate analysis			
			OR (95% CI)	*p*		OR (95% CI)	*p*	OR (95% CI)*	*p*

BMI									
<25	105 (72.9)	81 (79.4)	1						
25≤	39 (27.1)	21 (20.6)	1.43 (0.78–2.62)	0.24					
WC (cm)									
<85	46 (31.9)	53 (52.0)	1			1		1	
85≤	98 (68.1)	49 (48.0)	2.30 (1.37–3.89)	0.002		2.03 (1.09–3.75)	0.03	2.55 (1.32–4.93)	0.005
WHR									
<0.90	56 (38.9)	55 (53.9)	1			1		1	
0.90≤	88 (61.1)	47 (46.1)	1.84 (1.10–3.07)	0.02		1.27 (0.69–2.34)	0.44	0.85 (0.44–1.64)	0.63


b. Women									
Categorical variables	Individuals with APs (*n* = 87)	Individuals without APs (*n* = 127)	Univariate analysis			Multivariate analysis			
			OR (95% CI)	*p*		OR (95% CI)	*p*	OR (95% CI)^†^	*p*

BMI									
<25	60 (69.0)	116 (91.3)	1			1		1	
25≤	27 (31.0)	11 (8.7)	4.75 (2.20–10.2)	<0.0001		2.23 (0.89–5.56)	0.09	2.74 (1.05–7.13)	0.04
WC (cm)									
<90	55 (63.2)	115 (90.6)	1			1		1	
90≤	32 (36.8)	12 (9.4)	5.58 (2.67–11.7)	<0.0001		3.22 (1.28–8.12)	0.01	3.21 (1.23–8.40)	0.02
WHR									
<0.85	30 (34.5)	66 (52.0)	1			1		1	
0.85≤	57 (65.5)	61 (48.0)	2.06 (1.17–3.61)	0.01		1.28 (0.66–2.48)	0.47	0.77 (0.36–1.63)	0.49

*Adjusted for age (≥65 years) and alcohol drinking. ^†^Adjusted for age (≥65 years). AP, adenomatous polyp; BMI, body mass index; WC, waist circumference; WHR, waist-hip ratio.

**Table 6:** BMI and adenomatous polyps

Categorical variables	Men					Women			
	BMI ≥25 (*n* = 39)	BMI <25 (*n* = 105)	OR (95% CI)	*p*		BMI ≥25 (*n* = 27)	BMI <25 (*n* = 60)	OR (95% CI)	*p*
Number									
1	10 (25.6)	33 (31.4)	1			17 (63.0)	26 (43.3)	1	
2	10 (25.6)	23 (21.9)	1.44 (0.51–4.00)	0.49		4 (14.8)	20 (33.3)	0.31 (0.09–1.05)	0.06
≥3	19 (48.8)	49 (46.7)	1.28 (0.53–3.10)	0.58		6 (22.2)	14 (23.4)	0.66 (0.21–2.04)	0.46
Size (mm)									
<5	8 (20.5)	25 (23.8)	1			12 (44.4)	13 (21.7)	1	
≥5 <10	14 (35.9)	45 (42.9)	0.97 (0.36–2.64)	0.96		10 (37.0)	25 (41.7)	0.43 (0.15–1.27)	0.12
≥10	17 (43.6)	35 (33.3)	1.52 (0.57–4.06)	0.4		5 (18.6)	22 (36.6)	0.25 (0.07–0.86)	0.02
Location									
Proximal colon	21 (53.8)	48 (45.7)	1			15 (55.6)	27 (45.0)	1	
Distal colon	18 (46.2)	57 (54.3)	0.72 (0.35–1.51)	0.39		12 (44.4)	33 (55.0)	0.65 (0.26–1.63)	0.36
Advanced adenoma									
No	22 (56.4)	69 (65.7)	1			22 (81.4)	36 (60.0)	1	
Yes	17 (43.6)	36 (34.3)	1.48 (0.70–3.14)	0.3		5 (18.6)	24 (40.0)	0.34 (0.11–1.02)	0.05

BMI, body mass index.

**Table 7:** WC and adenomatous polyps

Categorical variables	Men					Women			
	WC ≥85 (*n* = 98)	WC <85 (*n* = 46)	OR (95% CI)	*p*		WC ≥90 (*n* = 32)	WC <90 (*n* = 55)	OR (95% CI)	*p*
Number									
1	25 (25.5)	18 (39.1)	1			15 (46.9)	28 (50.9)	1	
2	22 (22.4)	11 (23.9)	1.44 (0.56–3.70)	0.45		7 (21.9)	17 (30.9)	0.77 (0.26–2.27)	0.63
≥3	51 (52.1)	17 (37.0)	2.16 (0.95–4.89)	0.06		10 (31.2)	10 (18.2)	1.87 (0.64–5.49)	0.25
Size (mm)									
<5	23 (23.5)	10 (21.7)	1			10 (31.2)	15 (27.3)	1	
5< ≤10	40 (40.8)	19 (41.3)	0.92 (0.36–2.30)	0.85		16 (50.0)	19 (34.5)	1.26 (0.45–3.58)	0.66
10≤	35 (35.7)	17 (37.0)	0.90 (0.35–2.30)	0.82		6 (18.8)	21 (38.2)	0.43 (0.13–1.44)	0.17
Location									
Proximal colon	49 (50.0)	20 (43.5)	1			16 (50.0)	26 (47.3)	1	
Distal colon	49 (50.0)	26 (56.5)	0.77 (0.38–1.56)	0.47		16 (50.0)	29 (52.7)	0.90 (0.37–2.15)	0.81
Advanced adenoma									
No	62 (63.3)	29 (63.0)	1			25 (78.1)	33 (60.0)	1	
Yes	36 (36.7)	17 (37.0)	0.99 (0.48–2.05)	0.98		7 (21.9)	22 (40.0)	0.42 (0.16–1.14)	0.08

WC, waist circumference.

**Table 8:** WHR and adenomatous polyps

Categorical variables	Men					Women			
	WHR ≥0.9 (*n* = 88)	WHR <0.9 (*n* = 56)	OR (95% CI)	*p*		WHR ≥0.85 (*n* = 57)	WHR <0.85 (*n* = 30)	OR (95% CI)	*p*
Number									
1	23 (26.1)	20 (35.7)	1			29 (50.9)	14 (46.7)	1	
2	21 (23.9)	12 (21.4)	1.52 (0.60–3.85)	0.37		15 (26.3)	9 (30.0)	0.80 (0.28–2.29)	0.68
≥3	44 (50.0)	24 (42.9)	1.59 (0.73–3.47)	0.24		13 (22.8)	7 (23.3)	0.90 (0.29–2.75)	0.85
Size (mm)									
<5	19 (21.6)	14 (25.0)	1			17 (29.8)	8 (26.6)	1	
> 5 ≤10	37 (42.0)	22 (39.3)	1.24 (0.52–2.96)	0.63		24 (42.1)	11 (36.7)	1.03 (0.34–3.09)	0.96
≥10	32 (36.4)	20 (35.7)	1.18 (0.49–2.87)	0.72		16 (28.1)	11 (36.7)	0.68 (0.22–2.14)	0.51
Location									
Proximal colon	43 (48.9)	26 (46.4)	1			32 (56.1)	10 (33.3)	1	
Distal colon	45 (51.1)	30 (53.6)	0.91 (0.46–1.78)	0.78		25 (43.9)	20 (66.7)	0.39 (0.16–0.98)	0.04
Advanced adenoma									
No	55 (62.5)	36 (64.3)	1			40 (70.2)	18 (60.0)	1	
Yes	33 (37.5)	20 (35.7)	1.08 (0.54–2.17)	0.83		17 (29.8)	12 (40.0)	0.64 (0.25–1.61)	0.34

WHR, waist-hip ratio.

**Table 9:** Association between obesity and adipose tissue-related biomarkers in patients with adenomatous polyps

	Men					Women			
	Individuals with AP (*n* = 68)	Individuals without AP (*n* = 61)	OR (95% CI)	*p*		Individuals with AP (*n* = 48)	Individuals without AP (*n* = 76)	OR (95% CI)	*p*
Adiponectin (µm/ml)									
Quartile 1	17 (25.0)	15 (24.6)	1			10 (20.8)	21 (27.6)	1	
Quartile 2	18 (26.5)	15 (24.6)	1.06 (0.40–2.81)	0.91		13 (27.1)	18 (23.7)	1.52 (0.54–4.28)	0.43
Quartile 3	14 (20.6)	18 (29.5)	0.69 (0.26–1.84)	0.45		13 (27.1)	18 (23.7)	1.52 (0.54–4.28)	0.43
Quartile 4	19 (27.9)	13 (21.3)	1.29 (0.48–3.47)	0.61		12 (25.0)	19 (25.0)	1.33 (0.47–3.77)	0.6
*p* trend				0.84					0.62
Leptin (ng/ml)									
Quartile 1	12 (17.6)	20 (32.8)	1			11 (22.9)	20 (26.3)	1	
Quartile 2	18 (26.5)	15 (24.6)	2.00 (0.74–5.39)	0.17		6 (12.5)	25 (32.9)	0.44 (0.14–1.39)	0.15
Quartile 3	16 (23.5)	16 (26.2)	1.67 (0.62–4.51)	0.31		15 (31.3)	20 (26.3)	1.36 (0.50–3.69)	0.54
Quartile 4	22 (32.4)	10 (16.4)	3.67 (1.30–10.3)	0.01		16 (33.3)	11 (14.5)	2.65 (0.91–7.66)	0.07
*p* trend				0.02					0.02
HOMA-IR									
Quartile 1	15 (22.1)	18 (29.5)	1			11 (22.9)	20 (26.3)	1	
Quartile 2	18 (26.5)	14 (22.9)	1.54 (0.58–4.11)	0.38		10 (20.8)	21 (27.6)	0.87 (0.30–2.48)	0.79
Quartile 3	16 (23.5)	17 (27.9)	1.13 (0.43–2.97)	0.81		14 (29.2)	17 (22.4)	1.50 (0.54–4.16)	0.44
Quartile 4	19 (27.9)	12 (19.7)	1.90 (0.70–5.15)	0.2		13 (27.1)	18 (23.7)	1.31 (0.47–3.66)	0.6
*p* trend				0.32					0.41

AP, adenomatous polyp; HOMA-IR, homeostatic model assessment of insulin resistance.
