# Supplementary material for: Sex differences in associations among metabolic syndrome, obesity, related biomarkers, and colorectal adenomatous polyp risk in a Japanese population
Source: J Clin Biochem Nutr. 2018 Apr 3;63(2):154–63. doi: 10.3164/jcbn.18-11 (PMC6160732; doi:10.3164/jcbn.18-11)
Supplement: Supplemental Table 1 [file jcbn18-11st01.pdf]

Supplemental Table 1. Associations between obesity measures and adipokines or HOMA-IR analyzed by Pearson correlation coefficients

	Adiponectin	Leptin	HOMA-IR
BMI	p<0.0001, r = –0.36	p<0.0001, r = 0.61	p<0.0001, r = 0.32
WC	p<0.0001, r = –0.30	p<0.0001, r = 0.53	p<0.0001, r = 0.36
WHR	p = 0.001, r = –0.19	p<0.0001, r = 0.29	p<0.0001, r = 0.27

HOMA-IR, homeostatic model assessment of insulin resistance; BMI, body mass index; WC, waist circumference; WHR, waist-hip ratio.
