# Supplementary material for: Sex differences in associations among metabolic syndrome, obesity, related biomarkers, and colorectal adenomatous polyp risk in a Japanese population
Source: J Clin Biochem Nutr. 2018 Apr 3;63(2):154–63. doi: 10.3164/jcbn.18-11 (PMC6160732; doi:10.3164/jcbn.18-11)
Supplement: Supplemental Table 2 [file jcbn18-11st02.pdf]

Supplemental Table 2. Association between adipose tissue-related biomarkers and adenomatous polyps

a. Association with high-molecular-weight adiponectin

Categorical variables	Men					Women				
	High-molecular-weight adiponectin (mg/ml)					High-molecular-weight adiponectin (mg/ml)				
	Q1 (0.83–2.01) (*n* = 17)	Q2 (2.02–2.97) (*n* = 18)	Q3 (2.98–4.73) (*n* = 14)	Q4 (4.74–18.1) (*n* = 19)	*p*	Q1 (0.45–3.60) (*n* = 10)	Q2 (3.61–5.33) (*n* = 13)	Q3 (5.34–7.82) (*n* = 13)	Q4 (7.83–20.9) (*n* = 12)	*p*
Size (mm)										
<5	1 (5.9)	4 (22.3)	2 (14.3)	3 (15.8)		3 (30.0)	2 (15.4)	2 (15.4)	2 (16.7)	
≥5 <10	10 (58.8)	6 (33.3)	8 (57.1)	5 (26.3)		4 (40.0)	8 (61.5)	8 (61.5)	1 (8.3)	
≥10	6 (35.3)	8 (44.4)	4 (28.6)	11 (57.9)	0.34	3 (30.0)	3 (23.1)	3 (23.1)	9 (75.0)	0.06
Number										
1	3 (17.6)	7 (38.9)	2 (14.2)	5 (26.3)		4 (40.0)	7 (53.8)	8 (61.5)	6 (50.0)	
2	5 (29.4)	5 (27.8)	6 (42.9)	4 (21.1)		5 (50.0)	4 (30.8)	4 (30.8)	1 (8.3)	
≥3	9 (53.0)	6 (33.3)	6 (42.9)	10 (52.6)	0.57	1 (10.0)	2 (15.4)	1 (7.7)	5 (41.7)	0.2
Location										
Proximal colon	11 (64.7)	10 (55.6)	8 (57.1)	9 (47.4)		4 (40.0)	5 (38.5)	6 (46.2)	5 (41.7)	
Distal colon	6 (35.3)	8 (44.4)	6 (42.9)	10 (52.6)	0.78	6 (60.0)	8 (61.5)	7 (53.8)	7 (58.3)	0.98
Advanced adenoma										
No	11 (64.7)	9 (50.0)	10 (71.4)	8 (42.1)		7 (70.0)	10 (76.9)	10 (76.9)	3 (25.0)	
Yes	6 (35.3)	9 (50.0)	4 (28.6)	11 (57.9)	0.3	3 (30.0)	3 (23.1)	3 (23.1)	9 (75.0)	0.02

b. Association with leptin

Categorical variables	Men					Women				
	Leptin (ng/ml)					Leptin (ng/ml)				
	Q1 (2.0–4.1) (*n* = 12)	Q2 (4.2–6.2) (*n* = 18)	Q3 (6.3–9.3) (*n* = 16)	Q4 (9.4–129.0) (*n* = 22)	*p*	Q1 (2.2–6.8) (*n* = 11)	Q2 (6.9–10.0) (*n* = 6)	Q3 (10.1–18.6) (*n* = 15)	Q4 (18.7–76.0) (*n* = 16)	*p*
Size (mm)										
<5	1 (8.3)	2 (11.1)	3 (18.7)	4 (18.2)		3 (27.3)	0 (0.0)	2 (13.3)	4 (25.0)	
≥5 <10	5 (41.7)	10 (55.6)	5 (31.3)	9 (40.9)		3 (27.3)	3 (50.0)	7 (46.7)	8 (50.0)	
≥10	6 (50.0)	6 (33.3)	8 (50.0)	9 (40.9)	0.83	5 (45.4)	3 (50.0)	6 (40.0)	4 (25.0)	0.66
Number										
1	2 (16.7)	6 (33.4)	1 (6.2)	8 (36.4)		7 (63.6)	4 (66.6)	7 (46.7)	7 (43.7)	
2	3 (25.0)	4 (22.2)	5 (31.3)	8 (36.4)		2 (18.2)	1 (16.7)	6 (40.0)	5 (31.3)	
≥3	7 (58.3)	8 (44.4)	10 (62.5)	6 (27.2)	0.23	2 (18.2)	1 (16.7)	2 (13.3)	4 (25.0)	0.83
Location										
Proximal colon	7 (58.3)	9 (50.0)	10 (62.5)	12 (54.5)		5 (45.4)	2 (33.4)	7 (46.7)	6 (37.5)	
Distal colon	5 (41.7)	9 (50.0)	6 (37.5)	10 (45.5)	0.9	6 (54.6)	4 (66.6)	8 (53.3)	10 (62.5)	0.92
Advanced adenoma										
No	6 (50.0)	12 (66.7)	7 (43.8)	13 (59.1)		6 (54.6)	3 (50.0)	9 (60.0)	12 (75.0)	
Yes	6 (50.0)	6 (33.3)	9 (56.2)	9 (40.9)	0.56	5 (45.4)	3 (50.0)	6 (40.0)	4 (25.0)	0.61

c. Association with HOMA-IR

Categorical variables	Men					Women				
	HOMA-IR					HOMA-IR				
	Q1 (0.06–0.80) (*n* = 15)	Q2 (0.81–1.30) (*n* = 18)	Q3 (1.31–2.24) (*n* = 16)	Q4 (2.25–33.6) (*n* = 19)	*p*	Q1 (0.18–0.66) (*n* = 11)	Q2 (0.67–1.12) (*n* = 10)	Q3 (1.13–2.25) (*n* = 14)	Q4 (2.26–15.9) (*n* = 13)	*p*
Size (mm)										
<5	2 (13.3)	3 (16.7)	2 (12.6)	3 (15.8)		1 (9.1)	1 (10.0)	2 (14.2)	5 (38.5)	
≥5 <10	4 (26.7)	9 (50.0)	7 (43.7)	9 (47.4)		4 (36.4)	4 (40.0)	8 (57.3)	5 (38.5)	
≥10	9 (60.0)	6 (33.3)	7 (43.7)	7 (36.8)	0.82	6 (54.5)	5 (50.0)	4 (28.5)	3 (23.0)	0.33
Number										
1	3 (20.0)	5 (27.8)	2 (12.6)	7 (36.8)		6 (54.5)	6 (60.0)	6 (42.9)	7 (54.0)	
2	4 (26.7)	7 (38.9)	5 (31.1)	4 (21.1)		3 (27.2)	2 (20.0)	6 (42.9)	3 (23.0)	
≥3	8 (53.3)	6 (33.3)	9 (56.3)	8 (42.1)	0.6	2 (18.3)	2 (20.0)	2 (14.2)	3 (23.0)	0.92
Location										
Proximal colon	11 (73.3)	8 (44.4)	8 (50.0)	11 (57.9)		4 (36.4)	5 (50.0)	3 (21.4)	8 (61.5)	
Distal colon	4 (26.7)	10 (55.6)	8 (50.0)	8 (42.1)	0.38	7 (63.6)	5 (50.0)	11 (78.6)	5 (38.5)	0.18
Advanced adenoma										
No	6 (40.0)	11 (61.1)	9 (56.3)	12 (63.2)		5 (45.5)	5 (50.0)	10 (71.5)	10 (77.0)	
Yes	9 (60.0)	7 (38.9)	7 (43.7)	7 (36.8)	0.54	6 (54.5)	5 (50.0)	4 (28.5)	3 (23.0)	0.3

Q, quartile; HOMA-IR, homeostatic model assessment of insulin resistance.
